# Generalized Contractive Mappings and Weakly *α*-Admissible Pairs in *G*-Metric Spaces

**DOI:** 10.1155/2014/941086

**Published:** 2014-08-17

**Authors:** N. Hussain, V. Parvaneh, S. J. Hoseini Ghoncheh

**Affiliations:** ^1^Department of Mathematics, King Abdulaziz University, P.O. Box 80203, Jeddah 21589, Saudi Arabia; ^2^Department of Mathematics, Gilan-E-Gharb Branch, Islamic Azad University, Gilan-E-Gharb, Iran; ^3^Department of Mathematics, College of Science, Takestan Branch, Islamic Azad University, Takestan, Iran

## Abstract

The aim of this paper is to present some coincidence and common fixed point results for generalized (*ψ*, *φ*)-contractive mappings using partially weakly *G*-*α*-admissibility in the setup of *G*-metric space. As an application of our results, periodic points of weakly contractive mappings are obtained. We also derive certain new coincidence point and common fixed point theorems in partially ordered *G*-metric spaces. Moreover, some examples are provided here to illustrate the usability of the obtained results.

## 1. Introduction and Mathematical Preliminaries

The concept of a generalized metric space, or a *G*-metric space, was introduced by Mustafa and Sims [1]. In recent years, many authors have obtained different fixed point theorems for mappings satisfying various contractive conditions on *G*-metric spaces. For a survey of fixed point theory, its applications, different contractive conditions, and related topics in *G*-metric spaces we refer the reader to [1–33] and the references mentioned therein.

Recall that very recently Samet et al. [33] and Jleli and Samet [22] proved that several results in G-metric spaces can be deduced from the usual one. Later on, Agarwal and Karapnar [23] and Asadi et al. [25] suggested some new contraction mapping type to fail the approaches in [22, 33].

**Definition 1 (*G*-metric space [1]).**

Let *X* be a nonempty set and let *G* : *X* ^3^ → *R* ^+^ be a function satisfying the following properties:

(G1) *G*(*x*, *y*, *z*) = 0 if *x* = *y* = *z*;
(G2) 0 < *G*(*x*, *x*, *y*), for all *x*, *y* ∈ *X* with *x* ≠ *y*;
(G3) *G*(*x*, *x*, *y*) ≤ *G*(*x*, *y*, *z*), for all *x*, *y*, *z* ∈ *X* with *y* ≠ *z*;
(G4) *G*(*x*, *y*, *z*) = *G*(*x*, *z*, *y*) = *G*(*y*, *z*, *x*) = ⋯ (symmetry in all three variables);
(G5) *G*(*x*, *y*, *z*) ≤ *G*(*x*, *a*, *a*) + *G*(*a*, *y*, *z*), for all *x*, *y*, *z*, *a* ∈ *X* (rectangle inequality).

Then, the function *G* is called a *G*-metric on *X* and the pair (*X*, *G*) is called a *G*-metric space.

**Definition 2 (see [1]).**

Let (*X*, *G*) be a *G*-metric space and let {*x*_*n*_} be a sequence of points of *X*. A point *x* ∈ *X* is said to be the limit of the sequence {*x*_*n*_} if  lim⁡_*n*,*m*→*∞*_⁡*G*(*x*, *x*_*n*_, *x*_*m*_) = 0. In this case, one says that the sequence {*x*_*n*_} is *G*-convergent to *x*. Thus, if *x*_*n*_ → *x* in a *G*-metric space (*X*, *G*), then, for any *ɛ* > 0, there exists a positive integer *N* such that *G*(*x*, *x*_*n*_, *x*_*m*_) < *ɛ*, for all *n*, *m* ≥ *N*.

**Definition 3 (see [1]).**

Let (*X*, *G*) be a *G*-metric space. A sequence {*x*_*n*_} is called *G*-Cauchy if for every *ɛ* > 0, there is a positive integer *N* such that *G*(*x*_*n*_, *x*_*m*_, *x*_*l*_) < *ɛ*, for all *n*, *m*, *l* ≥ *N*; that is, if *G*(*x*_*n*_, *x*_*m*_, *x*_*l*_) → 0, as *n*, *m*, *l* → *∞*.

**Lemma 4 (see [1]).**

Let (*X*, *G*) be a *G*-metric space. Then, the following are equivalent:

1. {*x*_*n*_} is *G*-convergent to *x*;
2. *G*(*x*_*n*_, *x*_*n*_, *x*) → 0, as *n* → *∞*;
3. *G*(*x*_*n*_, *x*, *x*) → 0, as *n* → *∞*;
4. *G*(*x*_*m*_, *x*_*n*_, *x*) → 0, as *m*, *n* → *∞*.

**Lemma 5 (see [34]).**

If (*X*, *G*) is a *G*-metric space, then {*x*_*n*_} is a *G*-Cauchy sequence if and only if for every *ɛ* > 0, there exists a positive integer *N* such that *G*(*x*_*n*_, *x*_*m*_, *x*_*m*_) < *ɛ*, for all *m* > *n* ≥ *N*.

**Definition 6 (see [1]).**

A *G*-metric space (*X*, *G*) is said to be *G*-complete (or complete *G*-metric space) if every *G*-Cauchy sequence in (*X*, *G*) is *G*-convergent in *X*.

**Proposition 7 (see [1]).**

Let *X* be a *G*-metric space. Then for each *x*, *y*, *z*, *a* ∈ *X* it follows that

1. if *G*(*x*, *y*, *z*) = 0 then *x* = *y* = *z*,
2. *G*(*x*, *y*, *z*) ≤ *G*(*x*, *x*, *y*) + *G*(*x*, *x*, *z*),
3. *G*(*x*, *y*, *y*) ≤ 2*G*(*y*, *x*, *x*),
4. *G*(*x*, *y*, *z*) ≤ *G*(*x*, *a*, *z*) + *G*(*a*, *y*, *z*).

**Definition 8 (see [1]).**

Let (*X*, *G*) and (*X*′, *G*′) be two *G*-metric spaces. Then a function *f* : *X* → *X*′ is *G*-continuous at a point *x* ∈ *X* if and only if it is *G*-sequentially continuous at *x*; that is, whenever {*x*_*n*_} is *G*-convergent to *x*, {*f*(*x*_*n*_)} is *G*′-convergent to *f*(*x*).

The concept of an altering distance function was introduced by Khan et al. [35] as follows.

**Definition 9 .**

The function *ψ* : [0, *∞*)→[0, *∞*) is called an altering distance function, if the following properties are satisfied.

1. *ψ* is continuous and nondecreasing.
2. *ψ*(*t*) = 0 if and only if *t* = 0.

Samet et al. [36] defined the notion of *α*-admissible mappings in the framework of metric spaces as follows.

**Definition 10 .**

Let *T* be a self-mapping on *X* and let *α* : *X* × *X* → [0, *∞*) be a function. We say that *T* is an *α*-admissible mapping if

(1) $$\begin{array}{c} x,y\in X,\;\alpha \left(x,y\right)\geq 1⟹\alpha \left(Tx,Ty\right)\geq 1. \end{array}$$

For more details on *α*-admissible mappings we refer the reader to [37–39].

**Definition 11 (see [40]).**

Let (*X*, *G*) be a *G*-metric space and let *f* be a self-mapping on *X* and let *α* : *X* ^3^ → [0, *∞*) be a function. We say that *f* is a *G*-*α*-admissible mapping if

(2) $$\begin{array}{c} x,y,z\in X,\;\alpha \left(x,y,z\right)\geq 1⟹\alpha \left(fx,fy,fz\right)\geq 1. \end{array}$$

**Definition 12 .**

Let *X* be an arbitrary set, *α* : *X* × *X* × *X* → [0, *∞*), and *f* : *X* → *X*. A mapping *f* is called an *α*-dominating map on *X* if *α*(*x*, *fx*, *fx*) ≥ 1 or *α*(*x*, *x*, *fx*) ≥ 1 for each *x* in *X*.

**Example 13 .**

Let *X* = [0,1]. Let *f* : *X* → *X* be defined by *fx* = *x* ^1/3^ and let *α* : *X* × *X* × *X* → [0, *∞*) be defined by *α*(*x*, *y*, *z*) = *y* + *z* − 2*x* + 1. Then, *x* ≤ *x* ^1/3^ = *fx* for all *x* ∈ *X*. That is, 2*x* ^1/3^ − 2*x* + 1 ≥ 1. Thus, *f* is an *α*-dominating map.

**Definition 14 .**

Let (*X*, *G*) be a *G*-metric space. We say that *X* is *α*-regular if and only if the following hypothesis holds.

For any sequence {*x*_*n*_} in *X* with *α*(*x*_*n*_, *x*_*n*+1_, *x*_*n*+2_) ≥ 1 such that *x*_*n*_ → *z* as *n* → *∞*, it follows that *α*(*x*_*n*_, *z*, *z*) ≥ 1 or *α*(*z*, *x*_*n*_, *z*) ≥ 1 or *α*(*z*, *z*, *x*_*n*_) ≥ 1, for all *n* ∈ *N*.

**Definition 15 .**

Let *X* be a set and let *f*, *g* : *X* → *X* be given mappings. We say that the pair (*f*, *g*) is partially weakly *G*-*α*-admissible if and only if *α*(*fx*, *g* *fx*, *g* *fx*) ≥ 1 for all *x* ∈ *X*.

Let *X* be a nonempty set and *f* : *X* → *X* a given mapping. For every *x* ∈ *X*, let *f* ^−1^(*x*) = {*u* ∈ *X*∣*fu* = *x*}.

**Definition 16 .**

Let *X* be a set and let *f*, *g*, *R* : *X* → *X* be given mappings. We say that the pair (*f*, *g*) is partially weakly *G*-*α*-admissible with respect to *R* if and only if for all *x* ∈ *X*, *α*(*fx*, *gy*, *gy*) ≥ 1, where *y* ∈ *R* ^−1^(*fx*).

If *f* = *g*, we say that *f* is partially weakly *G*-*α*-admissible with respect to *R*.

If *R* = *I*_*X*_ (the identity mapping on *X*), then the previous definition reduces to the partially weakly *G*-*α*-admissible pair.

Following is an example of mappings *f*, *g*, *h*, *R*, *S*, and *T* for which ordered pairs (*f*, *g*), (*g*, *h*), and (*h*, *f*) are partially weakly *G*-*α*-admissible with respect to *R*, *S*, and *T*, respectively.

**Example 17 .**

Let *X* = [0, *∞*). We define functions *f*, *g*, *h*, *R*, *S*, *T* : *X* → *X* by

(3) $$\begin{array}{c} f\left(x\right)=\{\begin{array}{cc} x, & 0\leq x\leq 1,\\ 1, & 1\leq x\leq \infty , \end{array}\;\;g\left(x\right)=\{\begin{array}{cc} \sqrt{x}, & 0\leq x\leq 1,\\ 1, & 1\leq x\leq \infty , \end{array}\\ h\left(x\right)=\{\begin{array}{cc} x^{2}, & 0\leq x\leq 1,\\ 1, & 1\leq x\leq \infty , \end{array}\\ R\left(x\right)=\{\begin{array}{cc} x^{3}, & 0\leq x\leq 1,\\ 1, & 1\leq x\leq \infty , \end{array}\;\;S\left(x\right)=\{\begin{array}{cc} \sqrt[{4}]{x}, & 0\leq x\leq 1,\\ 1, & 1\leq x\leq \infty , \end{array}\\ T\left(x\right)=\{\begin{array}{cc} \sqrt[{3}]{x}, & 0\leq x\leq 1,\\ 1, & 1\leq x\leq \infty . \end{array} \end{array}$$

Also, let $\alpha (x,y,z)=1+\sinh^{-1}(\sqrt[{3}]{y+z-2x}/1+{\left(x+y+z\right)}^{4})$.

Jungck in [41] introduced the following definition.

**Definition 18 (see [42]).**

Let (*X*, *G*) be a *G*-metric space and let *f*, *g* : *X* → *X*. The pair (*f*, *g*) is said to be compatible if and only if lim⁡_*n*→*∞*_⁡*G*(*fgx*_*n*_, *fgx*_*n*_, *g* *fx*_*n*_) = 0, whenever {*x*_*n*_} is a sequence in *X* such that lim⁡_*n*→*∞*_⁡*fx*_*n*_ = lim⁡_*n*→*∞*_⁡*gx*_*n*_ = *t* for some *t* ∈ *X*.

The aim of this paper is to prove some coincidence and common fixed point theorems for nonlinear weakly (*ψ*, *φ*)-contractive mappings (*f*, *g*), (*g*, *h*), and (*h*, *f*) which are partially weakly *α*-admissible with respect to *R*, *S*, and *T*, respectively, in a *G*-metric space.

## 2. Main Results

Let (*X*, *G*) be a metric space and let *f*, *g*, *h*, *R*, *S*, *T* : *X* → *X* be six self-mappings. In the rest of this paper, unless otherwise stated, for all *x*, *y*, *z* ∈ *X*, let

(4) M(x,y,z)∈{G(Tx,Ry,Sz),  G(Tx,gy,gy)+G(Ry,hz,hz)+G(Sz,fx,fx)6,  G(Tx,Tx,gy)+G(Ry,Ry,hz)+G(Sz,Sz,fx)6,  G(Tx,fx,fx)+G(Ry,gy,gy)+G(Sz,Sz,hz)6,  G(Tx,Tx,fx)+G(Ry,Ry,gy)+G(Sz,hz,hz)6}.

From now on, let *α* : *X* ^3^ → [0, *∞*) be a function having the following property:

(5) $$\begin{array}{c} \text{If}\;\alpha \left(x,y,y\right)\geq 1\;\text{and}\;\alpha \left(y,z,w\right)\geq 1,\;\;\text{then}\;\alpha \left(x,y,w\right)\geq 1. \end{array}$$

For example, one can take *α* : [0, *∞*)^3^ → [0, *∞*) by *α*(*x*, *y*, *z*) = *e* ^2*x*−*y*−*z*^.

Our first result is the following.

**Theorem 19 .**

Let (*X*, *G*) be a *G*-complete *G*-metric space. Let *f*, *g*, *h*, *R*, *S*, *T* : *X* → *X* be six mappings such that *f*(*X*)⊆*R*(*X*), *g*(*X*)⊆*S*(*X*), and *h*(*X*)⊆*T*(*X*). Suppose that, for every three elements *x*, *y*, and *z* with *α*(*Tx*, *Ry*, *Sz*) ≥ 1, one has

(6) $$\begin{array}{c} \psi \left(G\left(fx,gy,hz\right)\right)\leq \psi \left(M\left(x,y,z\right)\right)-\varphi \left(M\left(x,y,z\right)\right), \end{array}$$

where *ψ*, *φ* : [0, *∞*)→[0, *∞*) are altering distance functions. Let *f*, *g*, *h*, *R*, *S*, and *T* be continuous, the pairs (*f*, *T*), (*g*, *R*), and (*h*, *S*) compatible, and the pairs (*f*, *g*), (*g*, *h*), and (*h*, *f*) partially weakly *α*-admissible with respect to *R*, *S*, and *T*, respectively. Then, the pairs (*f*, *T*), (*g*, *R*), and (*h*, *S*) have a coincidence point *z* in *X*. Moreover, if *α*(*Tz*, *Rz*, *Sz*) ≥ 1, then *z* is a coincidence point of *f*, *g*, *h*, *R*, *S*, and *T*.

**Proof**

Let *x*_0_ ∈ *X* be an arbitrary point. Since *f*(*X*)⊆*R*(*X*), we can choose *x*_1_ ∈ *X* such that *fx*_0_ = *Rx*_1_. Since *g*(*X*)⊆*S*(*X*), we can choose *x*_2_ ∈ *X* such that *gx*_1_ = *Sx*_2_. Also, as *h*(*X*)⊆*T*(*X*), we can choose *x*_3_ ∈ *X* such that *hx*_2_ = *Tx*_3_.

Continuing this process, we can construct a sequence {*z*_*n*_} defined by

(7) $$\begin{array}{c} z_{3n+1}=Rx_{3n+1}=fx_{3n},\\ z_{3n+2}=Sx_{3n+2}=gx_{3n+1},\\ z_{3n+3}=Tx_{3n+3}=hx_{3n+2} \end{array}$$

for all *n* ≥ 0.

Now, since *x*_1_ ∈ *R* ^−1^(*fx*_0_), *x*_2_ ∈ *S* ^−1^(*gx*_1_), and *x*_3_ ∈ *T* ^−1^(*hx*_2_) and (*f*, *g*), (*g*, *h*), and (*h*, *f*) are partially weakly *α*-admissible with respect to *R*, *S*, and *T*, respectively, we obtain that

(8) $$\begin{array}{c} \alpha \left(fx_{0}=Rx_{1},gx_{1}=Sx_{2},gx_{1}=Sx_{2}\right)\geq 1,\\ \alpha \left(gx_{1}=Sx_{2},hx_{2}=Tx_{3},hx_{2}=Tx_{3}\right)\geq 1,\\ \alpha \left(hx_{2}=Tx_{3},fx_{3}=Rx_{4},fx_{3}=Rx_{4}\right)\geq 1. \end{array}$$

Continuing this process, from (5), we get

(9) $$\begin{array}{c} \alpha \left(Tx_{3n},Rx_{3n+1},Sx_{3n+2}\right)\geq 1 \end{array}$$

for all *n* ∈ *N*.

Define *G*_*k*_ = *G*(*z*_*k*_, *z*_*k*+1_, *z*_*k*+2_). Suppose *G*_*k*_0__ = 0, for some *k*_0_. Then, *z*_*k*_0__ = *z*_*k*_0_+1_ = *z*_*k*_0_+2_. In the case that *k*_0_ = 3*n*, then *z*_3*n*_ = *z*_3*n*+1_ = *z*_3*n*+2_ gives *z*_3*n*+1_ = *z*_3*n*+2_ = *z*_3*n*+3_. Indeed,

(10) ψ(G(z3n+1,z3n+2,z3n+3)) =ψ(G(fx3n,gx3n+1,hx3n+2)) ≤ψ(M(x3n,x3n+1,x3n+2))−φ(M(x3n,x3n+1,x3n+2)).

If

(11) $$\begin{array}{c} M\left(x_{3n},x_{3n+1},x_{3n+2}\right)=G\left(Tx_{3n},Rx_{3n+1},Sx_{3n+2}\right), \end{array}$$

then

(12) $$\begin{array}{c} M\left(x_{3n},x_{3n+1},x_{3n+2}\right)=G\left(z_{3n},z_{3n+1},z_{3n+2}\right)=0. \end{array}$$

Thus,

(13) $$\begin{array}{c} \psi \left(G\left(z_{3n+1},z_{3n+2},z_{3n+3}\right)\right)\leq \psi \left(0\right)-\varphi \left(0\right), \end{array}$$

which implies that *z*_3*n*+1_ = *z*_3*n*+2_ = *z*_3*n*+3_.

Analogously, for other values of *M*(*x*_3*n*_, *x*_3*n*+1_, *x*_3*n*+2_), we can get this result.

Similarly, if *k*_0_ = 3*n* + 1, then *z*_3*n*+1_ = *z*_3*n*+2_ = *z*_3*n*+3_ gives *z*_3*n*+2_ = *z*_3*n*+3_ = *z*_3*n*+4_. Also, if *k*_0_ = 3*n* + 2, then *z*_3*n*+2_ = *z*_3*n*+3_ = *z*_3*n*+4_ implies that *z*_3*n*+3_ = *z*_3*n*+4_ = *z*_3*n*+5_. Consequently, *z*_*k*_0__ is a coincidence point of the pairs (*f*, *T*), (*g*, *R*), and (*h*, *S*). Indeed, let *k*_0_ = 3*n*. Then, we know that *z*_3*n*_ = *z*_3*n*+1_ = *z*_3*n*+2_ = *z*_3*n*+3_.

So,

(14) z3n=Tx3n=hx3n−1=z3n+1=Rx3n+1=fx3n=z3n+2=Sx3n+2=gx3n+1=z3n+3=Tx3n+3=hx3n+2.

This means that *T*(*x*_3*n*_) = *f*(*x*_3*n*_), *R*(*x*_3*n*+1_) = *g*(*x*_3*n*+1_), and *S*(*x*_3*n*+2_) = *h*(*x*_3*n*+2_).

On the other hand, the pairs (*f*, *T*), (*g*, *R*), and (*h*, *S*) are compatible. So, they are weakly compatible. Hence, *fT*(*x*_3*n*_) = *Tf*(*x*_3*n*_), *gR*(*x*_3*n*+1_) = *Rg*(*x*_3*n*+1_), and *hS*(*x*_3*n*+2_) = *Sh*(*x*_3*n*+2_) or, equivalently, *fz*_3*n*_ = *Tz*_3*n*+1_, *gz*_3*n*+1_ = *Rz*_3*n*+2_, and *hz*_3*n*+2_ = *Sz*_3*n*+3_.

Now, since *z*_3*n*_ = *z*_3*n*+1_ = *z*_3*n*+2_ = *z*_3*n*+3_, we have *fz*_3*n*_ = *Tz*_3*n*_, *gz*_3*n*_ = *Rz*_3*n*_, and *hz*_3*n*_ = *Sz*_3*n*_.

In the other cases, when *k*_0_ = 3*n* + 1 (*k*_0_ = 3*n* + 2), similarly, one can show that *z*_3*n*+1_ (*z*_3*n*+2_) is a coincidence point of the pairs (*f*, *T*), (*g*, *R*), and (*h*, *S*).

So, suppose that

(15) $$\begin{array}{c} G_{k}=G\left(z_{k},z_{k+1},z_{k+2}\right)>0 \end{array}$$

for each *k*; that is, *z*_*k*_ ≠ *z*_*k*+1_ for each *k*.

We complete the proof in three steps as follows.

*Step* *1*. We will prove that lim⁡_*k*→*∞*_⁡*G*(*z*_*k*_, *z*_*k*+1_, *z*_*k*+1_) = 0.

Since *α*(*Tx*_3*n*_, *Rx*_3*n*+1_, *Sx*_3*n*+2_) ≥ 1, using (6), we obtain that

(16) ψ(G(z3n+1,z3n+2,z3n+3)) =ψ(G(fx3n,gx3n+1,hx3n+2)) ≤ψ(M(x3n,x3n+1,x3n+2))−φ(M(x3n,x3n+1,x3n+2)) ≤ψ(M(x3n,x3n+1,x3n+2)).

Since *ψ* is a nondecreasing function, we get that

(17) $$\begin{array}{c} \begin{array}{ccc} G\left(z_{3n+1},z_{3n+2},z_{3n+3}\right)\leq M\left(x_{3n},x_{3n+1},x_{3n+2}\right). & & \end{array} \end{array}$$

If *M*(*x*_3*n*_, *x*_3*n*+1_, *x*_3*n*+2_) = *G*(*Tx*_3*n*_, *Rx*_3*n*+1_, *Sx*_3*n*+2_), then (17) becomes

(18) $$\begin{array}{c} \begin{array}{ccc} G\left(z_{3n+1},z_{3n+2},z_{3n+3}\right)\leq G\left(z_{3n},z_{3n+1},z_{3n+2}\right). & & \end{array} \end{array}$$

If

(19) M(x3n,x3n+1,x3n+2) =(G(Tx3n,gx3n+1,gx3n+1)+G(Rx3n+1,hx3n+2,hx3n+2)+G(Sx3n+2,fx3n,fx3n))/6,

then, from (G3) and (G4) in Definition 1,

(20) M(x3n,x3n+1,x3n+2) =(G(z3n,z3n+2,z3n+2)+G(z3n+1,z3n+3,z3n+3)+G(z3n+2,z3n+1,z3n+1))/6 ≤(G(z3n,z3n+1,z3n+2)+G(z3n+1,z3n+2,z3n+3)+G(z3n+1,z3n+2,z3n+3))/6,

and then (17) will be

(21) $$\begin{array}{c} \begin{array}{ccc} G\left(z_{3n+1},z_{3n+2},z_{3n+3}\right)\leq G\left(z_{3n},z_{3n+1},z_{3n+2}\right). & & \end{array} \end{array}$$

If

(22) M(x3n,x3n+1,x3n+2) =(G(Tx3n,Tx3n,gx3n+1)+G(Rx3n+1,Rx3n+1,hx3n+2)+G(Sx3n+2,Sx3n+2,fx3n))/6,

then, again from (G3) and (G4),

(23) M(x3n,x3n+1,x3n+2) =(G(z3n,z3n,z3n+2)+G(z3n+1,z3n+1,z3n+3)+G(z3n+2,z3n+2,z3n+1))/6 ≤(G(z3n,z3n+2,z3n+2)+G(z3n+1,z3n+3,z3n+3)+G(z3n+2,z3n+1,z3n+1))/3 ≤(G(z3n,z3n+1,z3n+2)+G(z3n+1,z3n+2,z3n+3)+G(z3n+1,z3n+2,z3n+3))/3,

and then (17) becomes

(24) $$\begin{array}{c} \begin{array}{ccc} G\left(z_{3n+1},z_{3n+2},z_{3n+3}\right)\leq G\left(z_{3n},z_{3n+1},z_{3n+2}\right). & & \end{array} \end{array}$$

If

(25) M(x3n,x3n+1,x3n+2) =(G(Tx3n,fx3n,fx3n)+G(Rx3n+1,gx3n+1,gx3n+1)+G(Sx3n+2,Sx3n+2,hx3n+2))/6,

then, again from (G3) and (G4),

(26) M(x3n,x3n+1,x3n+2) =(G(z3n,z3n+1,z3n+1)+G(z3n+1,z3n+2,z3n+2)+G(z3n+2,z3n+2,z3n+3))/6 ≤(2G(z3n,z3n,z3n+1)+2G(z3n+1,z3n+1,z3n+2)+2G(z3n+2,z3n+3,z3n+3))/6 ≤(2G(z3n,z3n+1,z3n+2)+2G(z3n+1,z3n+2,z3n+3)+2G(z3n+1,z3n+2,z3n+3))/6,

and then (17) becomes

(27) $$\begin{array}{c} \begin{array}{ccc} G\left(z_{3n+1},z_{3n+2},z_{3n+3}\right)\leq G\left(z_{3n},z_{3n+1},z_{3n+2}\right). & & \end{array} \end{array}$$

Finally, if

(28) M(x3n,x3n+1,x3n+2) =(G(Tx3n,Tx3n,fx3n)+G(Rx3n+1,Rx3n+1,gx3n+1)+G(Sx3n+2,hx3n+2,hx3n+2))/3,

then

(29) M(x3n,x3n+1,x3n+2) =(G(z3n,z3n,z3n+1)+G(z3n+1,z3n+1,z3n+2)+G(z3n+2,z3n+3,z3n+3))/6 ≤(G(z3n,z3n+1,z3n+2)+G(z3n+1,z3n+2,z3n+3)+G(z3n+1,z3n+2,z3n+3))/6,

and then (17) becomes

(30) $$\begin{array}{c} \begin{array}{ccc} G\left(z_{3n+1},z_{3n+2},z_{3n+3}\right)\leq G\left(z_{3n},z_{3n+1},z_{3n+2}\right). & & \end{array} \end{array}$$

Similarly it can be shown that

(31) $$\begin{array}{c} \begin{array}{ccc} G\left(z_{3n+2},z_{3n+3},z_{3n+4}\right)\leq G\left(z_{3n+1},z_{3n+2},z_{3n+3}\right), & & \end{array}\\ \begin{array}{ccc} G\left(z_{3n+3},z_{3n+4},z_{3n+5}\right)\leq G\left(z_{3n+2},z_{3n+3},z_{3n+4}\right). & & \end{array} \end{array}$$

Hence, we conclude that {*G*(*z*_*k*_, *z*_*k*+1_, *z*_*k*+2_)} is a nondecreasing sequence of nonnegative real numbers. Thus, there is an *r* ≥ 0 such that

(32) $$\begin{array}{c} \underset{k\to \infty }{\lim}G\left(z_{k},z_{k+1},z_{k+2}\right)=r. \end{array}$$

Reviewing the above argument, from (17), we have

(33) G(z3n+1,z3n+2,z3n+3) ≤M(x3n,x3n+1,x3n+2)≤G(z3n,z3n+1,z3n+2).

In general, we can show that

(34) $$\begin{array}{c} G\left(z_{k+1},z_{k+2},z_{k+3}\right)\leq M\left(x_{k},x_{k+1},x_{k+2}\right)\leq G\left(z_{k},z_{k+1},z_{k+2}\right). \end{array}$$

Letting *k* → *∞* in (34), we get that

(35) $$\begin{array}{c} \underset{k\to \infty }{\lim}M\left(x_{k},x_{k+1},x_{k+2}\right)=r. \end{array}$$

Letting *n* → *∞* and using (6), (35), and the continuity of *ψ* and *φ*, we get *ψ*(*r*) ≤ *ψ*(*r*) − *φ*(*r*), and hence *φ*(*r*) = 0. This gives us that

(36) $$\begin{array}{c} \underset{k\to \infty }{\lim}G\left(z_{k},z_{k+1},z_{k+2}\right)=0, \end{array}$$

from our assumptions about *φ*. Also, from Definition 1, part (G3), we have

(37) $$\begin{array}{c} \underset{k\to \infty }{\lim}G\left(z_{k},z_{k+1},z_{k+1}\right)=0. \end{array}$$

*Step* *2*. We will show that {*z*_*n*_} is a *G*-Cauchy sequence in *X*. So, we will show that, for every *ɛ* > 0, there exists *k* ∈ *N* such that, for all *m*, *n* ≥ *k*, *G*(*z*_*m*_, *z*_*n*_, *z*_*n*_) < *ɛ*.

Suppose the above statement is false. Then, there exists *ɛ* > 0 for which we can find subsequences {*z*_3*m*(*k*)_} and {*z*_3*n*(*k*)_} of {*z*_3*n*_} such that *n*(*k*) > *m*(*k*) ≥ *k* satisfying that

(38) $$\begin{array}{c} \begin{array}{ccc} G\left(z_{3m(k)},z_{3n(k)},z_{3n(k)}\right)\geq ɛ & & \end{array} \end{array}$$

and *n*(*k*) is the smallest number such that (38) holds; that is,

(39) $$\begin{array}{c} G\left(z_{3m(k)},z_{3n(k)-1},z_{3n(k)-1}\right)<ɛ. \end{array}$$

From rectangle inequality,

(40) G(z3m(k),z3n(k)+1,z3n(k)+2)≤G(z3m(k),z3n(k)−1,z3n(k)−1) +G(z3n(k)−1,z3n(k)+1,z3n(k)+2).

Hence, in (40), if *k* → *∞*, using (36) and (39), we have

(41) $$\begin{array}{c} \begin{array}{cc} \underset{k\to \infty }{\mathrm{limsup}}\;G\left(z_{3m(k)},z_{3n(k)+1},z_{3n(k)+2}\right)\leq ɛ. & \end{array} \end{array}$$

Also,

(42) G(z3m(k),z3n(k),z3n(k)) ≤G(z3m(k),z3n(k)+1,z3n(k)+1)+G(z3n(k)+1,z3n(k),z3n(k)) ≤G(z3m(k),z3n(k)+1,z3n(k)+2)+G(z3n(k)+1,z3n(k),z3n(k)).

Hence, in (42), if *k* → *∞*, using (36) and (38), we have

(43) $$\begin{array}{c} \begin{array}{cc} \underset{k\to \infty }{\mathrm{liminf}}\;G\left(z_{3m(k)},z_{3n(k)+1},z_{3n(k)+2}\right)\geq ɛ. & \end{array} \end{array}$$

On the other hand,

(44) $$\begin{array}{c} \begin{array}{cc} G\left(z_{3m(k)},z_{3n(k)+2},z_{3n(k)+2}\right)\leq G\left(z_{3m(k)},z_{3n(k)+1},z_{3n(k)+2}\right). & \end{array} \end{array}$$

Hence, in (44), if *k* → *∞*, from (43), we have

(45) $$\begin{array}{c} \underset{k\to \infty }{\mathrm{limsup}}\;G\left(z_{3m(k)},z_{3n(k)+2},z_{3n(k)+2}\right)\leq ɛ. \end{array}$$

Also,

(46) G(z3m(k),z3n(k),z3n(k)) ≤G(z3m(k),z3n(k)+2,z3n(k)+2)+G(z3n(k)+2,z3n(k),z3n(k)).

Hence, in (46), if *k* → *∞*, using (36) and (38), we have

(47) $$\begin{array}{c} \underset{k\to \infty }{\mathrm{liminf}}\;G\left(z_{3m(k)},z_{3n(k)+2},z_{3n(k)+2}\right)\geq ɛ. \end{array}$$

In a similar way, we have

(48) G(z3m(k)+1,z3m(k)+1,z3n(k)+2)≤2G(z3m(k)+1,z3n(k)+2,z3n(k)+2)≤2G(z3m(k)+1,z3m(k),z3m(k))+2G(z3m(k),z3n(k)+2,z3n(k)+2).

Therefore, from (48) by taking limit when *k* → *∞*, using (36) and (45), we get that

(49) $$\begin{array}{c} \underset{k\to \infty }{\mathrm{limsup}}\;G\left(z_{3m(k)+1},z_{3m(k)+1},z_{3n(k)+2}\right)\leq 2ɛ. \end{array}$$

Further, we can obtain that

(50) $$\begin{array}{c} \begin{array}{cc} \underset{k\to \infty }{\mathrm{limsup}}\;G\left(z_{3m(k)+1},z_{3n(k)+2},z_{3n(k)+2}\right)\leq 4ɛ, & \end{array}\\ \begin{array}{cc} \underset{k\to \infty }{\mathrm{limsup}}\;G\left(z_{3m(k)},z_{3m(k)},z_{3n(k)+2}\right)\leq 2ɛ. & \end{array} \end{array}$$

Also,

(51) liminf⁡k→∞ 2G(z3m(k),z3n(k)+2,z3n(k)+2) ≥liminf⁡k→∞ G(z3m(k),z3n(k)+2,z3n(k)+2)≥ɛ,

or, equivalently,

(52) $$\begin{array}{c} \begin{array}{cc} \underset{k\to \infty }{\mathrm{liminf}}\;G\left(z_{3m(k)},z_{3m(k)},z_{3n(k)+2}\right)\geq \frac{ɛ}{2}. & \end{array} \end{array}$$

Also,

(53) G(z3m(k),z3n(k),z3n(k))≤G(z3m(k),z3m(k)+1,z3m(k)+1)+G(z3m(k)+1,z3n(k),z3n(k))≤G(z3m(k),z3m(k)+1,z3m(k)+1)+G(z3m(k)+1,z3n(k)+2,z3n(k)+2) +G(z3n(k)+2,z3n(k),z3n(k))≤G(z3m(k),z3m(k)+1,z3m(k)+1)+G(z3m(k)+1,z3n(k)+2,z3n(k)+3) +G(z3n(k)+2,z3n(k),z3n(k)).

Hence, in (53), if *k* → *∞*, using (36) and (38), we have

(54) $$\begin{array}{c} \begin{array}{cc} \underset{k\to \infty }{\mathrm{limsup}}\;G\left(z_{3m(k)+1},z_{3n(k)+2},z_{3n(k)+3}\right)\geq ɛ. & \end{array} \end{array}$$

Since *α*(*Tx*_3*m*(*k*)_, *Rx*_3*n*(*k*)+1_, *Sx*_3*n*(*k*)+2_) ≥ 1, putting *x* = *x*_3*m*(*k*)_, *y* = *x*_3*n*(*k*)+1_, and *z* = *x*_3*n*(*k*)+2_ in (6), for all *k* ≥ 0, we have

(55) ψ(G(z3m(k)+1,z3n(k)+2,z3n(k)+3)) =ψ(G(fx3m(k),gx3n(k)+1,hx3n(k)+2)) ≤ψ(M(x3m(k),x3n(k)+1,x3n(k)+2))  −φ(M(x3m(k),x3n(k)+1,x3n(k)+2)),

where

(56) M(x3m(k),x3n(k)+1,x3n(k)+2) ∈{G(Tx3m(k),Rx3n(k)+1,Sx3n(k)+2),(G(Tx3m(k),gx3n(k)+1,gx3n(k)+1)+G(Rx3n(k)+1,hx3n(k)+2,hx3n(k)+2)+G(Sx3n(k)+2,fx3m(k),fx3m(k)))/6,(G(Tx3m(k),Tx3m(k),gx3n(k)+1)+G(Rx3n(k)+1,Rx3n(k)+1,hx3n(k)+2)+G(Sx3n(k)+2,Sx3n(k)+2,fx3m(k)))/6,(G(Tx3m(k),fx3m(k),fx3m(k))+G(Rx3n(k)+1,gx3n(k)+1,gx3n(k)+1)+G(Sx3n(k)+2,Sx3n(k)+2,hx3n(k)+2))/6,(G(Tx3m(k),Tx3m(k),fx3m(k))+G(Rx3n(k)+1,Rx3n(k)+1,gx3n(k)+1)+G(Sx3n(k)+2,hx3n(k)+2,hx3n(k)+2))/6} ={G(z3m(k),z3n(k)+1,z3n(k)+2),(G(z3m(k),z3n(k)+2,z3n(k)+2)+G(z3n(k)+1,z3n(k)+3,z3n(k)+3)+G(z3n(k)+2,z3m(k)+1,z3m(k)+1))/6,(G(z3m(k),z3m(k),z3n(k)+2)+G(z3n(k)+1,z3n(k)+1,z3n(k)+3)+G(z3n(k)+2,z3n(k)+2,z3m(k)+1))/6,(G(z3m(k),z3m(k)+1,z3m(k)+1)+G(z3n(k)+1,z3n(k)+2,z3n(k)+2)+G(z3n(k)+2,z3n(k)+2,z3n(k)+3))/6,(G(z3m(k),z3m(k),z3m(k)+1)+G(z3n(k)+1,z3n(k)+1,z3n(k)+2)+G(z3n(k)+2,z3n(k)+3,z3n(k)+3))/6}.

If *M*(*x*_3*m*(*k*)_, *x*_3*n*(*k*)+1_, *x*_3*n*(*k*)+2_) = *G*(*z*_3*m*(*k*)_, *z*_3*n*(*k*)+1_, *z*_3*n*(*k*)+2_), from (54) and (41), if *k* → *∞* in (55), we have

(57) ψ(ɛ)≤ψ(limsup⁡k→∞ G(z3m(k)+1,z3n(k)+2,z3n(k)+3))≤ψ(ɛ)−φ(liminf⁡k→∞ G(z3m(k),z3n(k)+1,z3n(k)+2)),

which is a contradiction to (43).

If

(58) M(x3m(k),x3n(k)+1,x3n(k)+2)=(G(z3m(k),z3n(k)+2,z3n(k)+2)+G(z3n(k)+1,z3n(k)+3,z3n(k)+3)+G(z3n(k)+2,z3m(k)+1,z3m(k)+1))/6,

from (37), (45), (49), and (54), if *k* → *∞* in (55), we have

(59) ψ(ɛ) ≤ψ(ɛ+2ɛ3)−φ(liminf⁡k→∞ M(x3m(k),x3n(k)+1,x3n(k)+2)),

which is a contradiction to (47).

If

(60) M(x3m(k),x3n(k)+1,x3n(k)+2)=(G(z3m(k),z3m(k),z3n(k)+2)+G(z3n(k)+1,z3n(k)+1,z3n(k)+3)+G(z3n(k)+2,z3n(k)+2,z3m(k)+1))/6,

from (37), (50), and (54), if *k* → *∞* in (55), we have

(61) $$\begin{array}{c} \psi \left(ɛ\right)\leq \psi \left(\frac{ɛ}{2}\right)-\varphi \left(\underset{k\to \infty }{\mathrm{liminf}}\;M\left(x_{3m(k)},x_{3n(k)+1},x_{3n(k)+2}\right)\right), \end{array}$$

which is a contradiction to (52).

If

(62) M(x3m(k),x3n(k)+1,x3n(k)+2) =(G(z3m(k),z3m(k)+1,z3m(k)+1)+G(z3n(k)+1,z3n(k)+2,z3n(k)+2)+G(z3n(k)+2,z3n(k)+2,z3n(k)+3))/6,

from (37) and (54), if *k* → *∞* in (55), we have

(63) $$\begin{array}{c} \begin{array}{cc} \psi \left(ɛ\right)\leq \psi \left(0\right)-\varphi \left(0\right)=0. & \end{array} \end{array}$$

If

(64) M(x3m(k),x3n(k),x3n(k)+1)=(G(z3m(k),z3m(k),z3m(k)+1)+G(z3n(k)+1,z3n(k)+1,z3n(k)+2)+G(z3n(k)+2,z3n(k)+3,z3n(k)+3))/6,

from (37) and (54), if *k* → *∞* in (55), we have

(65) $$\begin{array}{c} \begin{array}{cc} \psi \left(ɛ\right)\leq \psi \left(0\right)-\varphi \left(0\right)=0. & \end{array} \end{array}$$

Hence, (63) and (65) yield that *ɛ* = 0 which is a contradiction. Consequently, {*z*_*n*_} is a *G*-Cauchy sequence.

*Step* *3*. We will show that *f*, *g*, *h*, *R*, *S*, and *T* have a coincidence point.

Since {*z*_*n*_} is a *G*-Cauchy sequence in the complete *G*-metric space *X*, there exists *z* ∈ *X* such that

(66) lim⁡n→∞G(z3n+1,z3n+1,z) =lim⁡n→∞G(Rx3n+1,Rx3n+1,z)=lim⁡n→∞G(fx3n,fx3n,z)=0,lim⁡n→∞G(z3n+2,z3n+2,z) =lim⁡n→∞G(Sx3n+2,Sx3n+2,z) =lim⁡n→∞G(gx3n+1,gx3n+1,z)=0,lim⁡n→∞G(z3n+3,z3n+3,z) =lim⁡n→∞G(Tx3n+3,Tx3n+3,z) =lim⁡n→∞G(hx3n+2,hx3n+2,z)=0.

Hence,

(67) $$\begin{array}{c} Tx_{3n}⟶z,\;fx_{3n}⟶z,\;\text{as}\;\;n⟶\infty . \end{array}$$

As (*f*, *T*) is compatible, so

(68) $$\begin{array}{c} \begin{array}{cc} \underset{n\to \infty }{\lim}G\left(Tfx_{3n},fTx_{3n},fTx_{3n}\right)=0. & \end{array} \end{array}$$

Moreover, from lim⁡_*n*→*∞*_⁡*G*(*fx*_3*n*_, *fx*_3*n*_, *z*) = 0, lim⁡_*n*→*∞*_ *G*(*Tx*_3*n*_, *z*, *z*) = 0, and the continuity of *T* and *f*, we obtain

(69) $$\begin{array}{c} \begin{array}{cc} \underset{n\to \infty }{\lim}G\left(Tfx_{3n},Tfx_{3n},Tz\right)=0=\underset{n\to \infty }{\lim}G\left(fTx_{3n},fz,fz\right). & \end{array} \end{array}$$

By the rectangle inequality, we have

(70) G(Tz,fz,fz) ≤G(Tz,Tfx3n,Tfx3n)+G(Tfx3n,fz,fz) ≤G(Tz,Tfx3n,Tfx3n)+G(Tfx3n,fTx3n,fTx3n)  +G(fTx3n,fz,fz).

Taking limit as *n* → *∞* in (70), using (68) and (69), we obtain

(71) $$\begin{array}{c} G\left(Tz,fz,fz\right)\leq 0, \end{array}$$

which implies that *fz* = *Tz*; that is, *z* is a coincidence point of *f* and *T*.

Similarly, we can obtain that *gz* = *Rz* and *hz* = *Sz*.

Now, let *α*(*Tz*, *Rz*, *Sz*) ≥ 1. By (6), we have

(72) $$\begin{array}{c} \begin{array}{cc} \psi \left(G\left(fz,gz,hz\right)\right)\leq \psi \left(M\left(z,z,z\right)\right)-\varphi \left(M\left(z,z,z\right)\right), & \end{array} \end{array}$$

where

(73) M(z,z,z) ∈{G(Tz,Rz,Sz),   G(Tz,gz,gz)+G(Rz,hz,hz)+G(Sz,fz,fz)6,   G(Tz,Tz,gz)+G(Rz,Rz,hz)+G(Sz,Sz,fz)6,   G(Tz,fz,fz)+G(Rz,gz,gz)+G(Sz,Sz,hz)6,   G(Tz,Tz,fz)+G(Rz,Rz,gz)+G(Sz,hz,hz)6}.

Let *G*(*fz*, *gz*, *hz*) > 0; that is, *fz* ≠ *gz* = *hz* or *fz* = *gz* ≠ *hz*.

If *M*(*z*, *z*, *z*) = *G*(*Tz*, *Rz*, *Sz*) = *G*(*fz*, *gz*, *hz*), from (72), we have

(74) $$\begin{array}{c} \psi \left(G\left(fz,gz,hz\right)\right)\leq \psi \left(G\left(fz,gz,hz\right)\right)-\varphi \left(G\left(fz,gz,hz\right)\right); \end{array}$$

hence, *G*(*fz*, *gz*, *hz*) = 0, a contradiction.

If *M*(*z*, *z*, *z*) = (*G*(*Tz*, *gz*, *gz*) + *G*(*Rz*, *hz*, *hz*) + *G*(*Sz*, *fz*, *fz*))/6 and *fz* ≠ *gz* = *hz*, then

(75) M(z,z,z) ≤G(fz,gz,gz)+G(gz,hz,hz)+2G(hz,hz,fz)6 =3G(hz,hz,fz)6≤3G(fz,gz,hz)6,

so, from (72), we have

(76) ψ(G(fz,gz,hz)) ≤ψ(G(fz,gz,hz)2)  −φ(G(fz,gz,gz)+0+G(hz,fz,fz)6);

that is,

(77) φ(G(fz,gz,gz)+0+G(hz,fz,fz)6) ≤ψ(G(iz,gz,hz)2)−ψ(G(fz,gz,hz))≤0;

hence, *fz* = *gz* = *hz*, a contradiction to *G*(*fz* = *gz* = *hz*) > 0.

In the other cases, by a similar manner, we can show that *fz* = *gz* = *hz* = *Tz* = *Rz* = *Sz*.

In the following theorem, we will omit the compatibility and continuity assumptions.

**Theorem 20 .**

Let (*X*, *G*) be an *α*-regular *G*-metric space and *f*, *g*, *h*, *R*, *S*, *T* : *X* → *X* six mappings such that *f*(*X*)⊆*R*(*X*), *g*(*X*)⊆*S*(*X*), and *h*(*X*)⊆*T*(*X*) and *RX*, *SX*, and *TX* are *G*-complete subsets of *X*. Suppose that, for elements *x*, *y*, and *z* with *α*(*Tx*, *Ry*, *Sz*) ≥ 1, we have

(78) $$\begin{array}{c} \begin{array}{cc} \psi \left(G\left(fx,gy,hz\right)\right)\leq \psi \left(M\left(x,y,z\right)\right)-\varphi \left(M\left(x,y,z\right)\right), & \end{array} \end{array}$$

where *ψ*, *φ* : [0, *∞*)→[0, *∞*) are altering distance functions. Then, the pairs (*f*, *T*), (*g*, *R*), and (*h*, *S*) have a coincidence point *z* in *X* provided that the pairs (*f*, *T*), (*g*, *R*), and (*h*, *S*) are weakly compatible and the pairs (*f*, *g*), (*g*, *h*), and (*h*, *f*) are partially weakly *α*-admissible with respect to *R*, *S*, and *T*, respectively. Moreover, if *α*(*Tz*, *Rz*, *Sz*) ≥ 1, then *z* ∈ *X* is a coincidence point of *f*, *g*, *h*, *R*, *S*, and *T*.

**Proof**

Following the proof of Theorem 19, there exists *z* ∈ *X* such that

(79) $$\begin{array}{c} \begin{array}{cc} \underset{k\to \infty }{\lim}G\left(z_{k},z_{k},z\right)=0. & \end{array} \end{array}$$

Since *R*(*X*) is *G*-complete and {*z*_3*n*+1_}⊆*R*(*X*), therefore *z* ∈ *R*(*X*), so there exists *u* ∈ *X* such that *z* = *Ru* and

(80) $$\begin{array}{c} \underset{n\to \infty }{\lim}G\left(z_{3n+1},z_{3n+1},Ru\right)=\underset{n\to \infty }{\lim}G\left(Rx_{3n+1},Rx_{3n+1},Ru\right)=0. \end{array}$$

Similarly, there exists *v*, *w* ∈ *X* such that *z* = *Sv* = *Tw* and

(81) $$\begin{array}{c} \underset{n\to \infty }{\lim}G\left(Sx_{3n+2},Sx_{3n+2},Sv\right)=\underset{n\to \infty }{\lim}G\left(Tx_{3n},Tx_{3n},Tw\right)=0. \end{array}$$

Now we prove that *w* is a coincidence point of *f* and *T*.

As *Tx*_3*n*_ → *z* = *Tw* = *Ru* = *Sv* as *n* → *∞*, *α*-regularity of *X* implies that *α*(*Tx*_3*n*_, *Ru*, *Sv*) ≥ 1. Therefore, from (6), we have

(82) $$\begin{array}{c} \psi \left(G\left(fx_{3n},gu,hv\right)\right)\leq \psi \left(M\left(x_{3n},u,v\right)\right)-\varphi \left(M\left(x_{3n},u,v\right)\right), \end{array}$$

where

(83) M(x3n,u,v)∈{G(Tx3n,Ru,Sv),  G(Tx3n,gu,gu)+G(Ru,hv,hv)+G(Sv,fx3n,fx3n)6,  G(Tx3n,Tx3n,gu)+G(Ru,Ru,hv)+G(Sv,Sv,fx3n)6,  G(Tx3n,fx3n,fx3n)+G(Ru,gu,gu)+G(Sv,Sv,hv)6,  G(Tx3n,Tx3n,fx3n)+G(Ru,Ru,gu)+G(Sv,hv,hv)6}.

Let *M*(*x*_3*n*_, *u*, *v*) = *G*(*Tx*_3*n*_, *Ru*, *Sv*).

Letting *n* → *∞* in (82), from the continuity of *ψ* and *φ*, we get

(84) $$\begin{array}{c} \begin{array}{ccc} \psi \left(G\left(z,gu,hv\right)\right)\leq \psi \left(0\right)-\varphi \left(0\right)=0, & & \end{array} \end{array}$$

so *Tw* = *Ru* = *Sv* = *z* = *gu* = *hv*.

As *g* and *R* are weakly compatible, we have *gz* = *gR* *u* = *Rg* *u* = *Rz*. Thus *z* is a coincidence point of *f* and *T*.

Similarly, in other cases for *M*(*x*, *y*, *z*), it can be shown that *z* is a coincidence point of the pairs (*g*, *R*) and (*h*, *S*).

The rest of the proof is similar to the proof of Theorem 19.

Assume that

(85) M1(x,y,z) ∈{G(x,y,z),   G(x,gy,gy)+G(y,hz,hz)+G(z,fx,fx)6,   G(x,x,gy)+G(y,y,hz)+G(z,z,fx)6,   G(x,fx,fx)+G(y,gy,gy)+G(z,z,hz)6,   G(x,x,fx)+G(y,y,gy)+G(z,hz,hz)6}.

Taking *R* = *S* = *T* = *I*_*X*_ (the identity mapping on *X*) in the previous theorems, we obtain the following common fixed point result.

**Corollary 21 .**

Let (*X*, *G*) be a *G*-complete *G*-metric space. Let *f*, *g*, *h* : *X* → *X* be three mappings. Suppose that, for every three elements *x*, *y*, and *z* with *α*(*x*, *y*, *z*) ≥ 1, we have

(86) $$\begin{array}{c} \psi \left(G\left(fx,gy,hz\right)\right)\leq \psi \left(M_{1}\left(x,y,z\right)\right)-\varphi \left(M_{1}\left(x,y,z\right)\right), \end{array}$$

where *ψ*, *φ* : [0, *∞*)→[0, *∞*) are altering distance functions. Let the pairs (*f*, *g*), (*g*, *h*), and (*h*, *f*) be partially weakly *α*-admissible. Then, the triple (*f*, *g*, *h*) has a common fixed point *z* in *X* provided that (a) *f*, *g*, and *h* are continuous or (b) *X* is *α*-regular.

Assume that

(87) M3(x,y,z) ∈{G(Rx,Ry,Rz),   G(Rx,fy,fy)+G(Ry,fz,fz)+G(Rz,fx,fx)3,   G(Rx,Rx,fy)+G(Ry,Ry,fz)+G(Rz,Rz,fx)3,   G(Rx,fx,fx)+G(Ry,fy,fy)+G(Rz,fz,fz)3,   G(Rx,Rx,fx)+G(Ry,Ry,fy)+G(Rz,Rz,fz)3}.

Taking *f* = *g* = *h* in Theorems 19 and 20, we obtain the following coincidence point result.

**Corollary 22 .**

Let (*X*, *G*) be a *G*-metric space. Let *f*, *R* : *X* → *X* be two mappings such that *f*(*X*)⊆*R*(*X*). Suppose that, for every three elements *x*, *y*, and *z* with *α*(*Rx*, *Ry*, *Rz*) ≥ 1, we have

(88) $$\begin{array}{c} \psi \left(G\left(fx,fy,fz\right)\right)\leq \psi \left(M_{3}\left(x,y,z\right)\right)-\varphi \left(M_{3}\left(x,y,z\right)\right), \end{array}$$

where *ψ*, *φ* : [0, *∞*)→[0, *∞*) are altering distance functions. Let the pair (*f*, *R*) be compatible and *f* is partially weakly *α*-admissible w.r.t. *R*. Then, (*f*, *R*) has a coincidence point *z* in *X* provided that (a) *f* and *R* are continuous and (*X*, *G*) is a *G*-complete *G*-metric space or (b) *X* is *α*-regular and *R*(*X*) is *G*-complete.

**Theorem 23 .**

Under the hypotheses of Corollary 22, *f* and *R* have a common fixed point in *X* if *R* is an *α*-dominating map. Moreover, *f* and *R* have one and only one common fixed point if *α*(*u*, *u*, *v*) ≥ 1 or *α*(*u*, *v*, *v*) ≥ 1, where *u* and *v* are common fixed points of *f* and *R*.

**Proof**

Corollary 22 guarantees that there is a *z* ∈ *X* such that *fz* = *Rz*. Since *f* and *R* are weakly compatible (since the pair (*f*, *R*) is compatible), we have *f* *Rz* = *R* *fz*. Let *w* = *Rz* = *fz*. Therefore, we have

(89) $$\begin{array}{c} fw=Rw. \end{array}$$

Since *R* is an *α*-dominating map,

(90) $$\begin{array}{c} \alpha \left(w,w,Rw\right)=\alpha \left(Rz,Rz,Rw\right)\geq 1. \end{array}$$

If *z* = *w*, then *z* is a common fixed point of *f* and *R*. If *z* ≠ *w*, then, from (90) *α*(*Rz*, *Rz*, *Rw*) ≥ 1, from (88), we have

(91) $$\begin{array}{c} \psi \left(G\left(fz,fz,fw\right)\right)\leq \psi \left(M_{3}\left(z,z,w\right)\right)-\varphi \left(M_{3}\left(z,z,w\right)\right), \end{array}$$

where

(92) M3(z,z,w) ∈{G(Rz,Rz,Rw),   G(Rz,fz,fz)+G(Rz,fw,fw)+G(Rw,fz,fz)6,   G(Rz,Rz,fz)+G(Rz,Rz,fw)+G(Rw,Rw,fz)6,   G(Rz,fz,fz)+G(Rz,fz,fz)+G(Rw,Rw,fw)6,   G(Rz,Rz,fz)+G(Rz,Rz,fz)+G(Rw,fw,fw)6}.

Let *M*_3_(*z*, *z*, *w*) = (*G*(*Rz*, *fz*, *fz*) + *G*(*Rz*, *fw*, *fw*) + *G*(*Rw*, *fz*, *fz*))/6. Then, from (91),

(93) ψ(G(fz,fz,fw))≤ψ(G(Rz,fz,fz)+G(Rz,fw,fw)+G(Rw,fz,fz)6) −φ(G(Rz,fz,fz)+G(Rz,fw,fw)+G(Rw,fz,fz)6)=ψ(G(fz,fz,fz)+G(fz,fw,fw)+G(fw,fz,fz)6) −φ(G(fz,fz,fz)+G(fz,fw,fw)+G(fw,fz,fz)6)≤ψ(2G(fz,fz,fw)+G(fz,fz,fw)6) −φ(G(fz,fz,fz)+G(fz,fw,fw)+G(fw,fz,fz)6).

Therefore, *φ*((*G*(*fz*, *fz*, *fz*) + *G*(*fz*, *fw*, *fw*) + *G*(*fw*, *fz*, *fz*))/6) = 0. So, *fz* = *fw*. Now, since *w* = *Rz* = *fz* and *fw* = *Rw*, we have *w* = *Rw* = *fw*.

Suppose that *α*(*u*, *u*, *v*) ≥ 1 or *α*(*u*, *v*, *v*) ≥ 1, where *u* and *v* are common fixed points of *f* and *R*. We claim that common fixed point of *f* and *R* is unique. Assume on the contrary that *fu* = *Ru* = *u* and *fv* = *Rv* = *v* and *u* ≠ *v*. Without any loss of generality, we may assume that *α*(*u*, *u*, *v*) = *α*(*Ru*, *Ru*, *Rv*) ≥ 1. Using (88), we obtain

(94) ψ(G(u,u,v)) =ψ(G(fu,fu,fv))≤ψ(M3(u,u,v))−φ(M3(u,u,v)).

Let *M*_3_(*u*, *u*, *v*) = (*G*(*Ru*, *fu*, *fu*) + *G*(*Ru*, *fv*, *fv*) + *G*(*Rv*, *fu*, *fu*))/6. Then we have

(95) ψ(G(u,u,v))≤ψ(G(Ru,fu,fu)+G(Ru,fv,fv)+G(Rv,fu,fu)6) −φ(G(Ru,fu,fu)+G(Ru,fv,fv)+G(Rv,fu,fu)6)≤ψ(2G(v,u,u)+G(v,u,u)6) −φ(G(u,u,u)+G(u,v,v)+G(v,u,u)6).

Therefore, *u* = *v*, a contradiction.

In the other cases the proof will be done in a similar way.

**Example 24 .**

Let *X* = [0, *∞*), *G* on *X* given by *G*(*x*, *y*, *z*) = |*x* − *y*| + |*y* − *z*| + |*z* − *x*|, for all *x*, *y*, *z* ∈ *X*, and *α* : *X* ^3^ → [0, *∞*) given by *α*(*x*, *y*, *z*) = *e* ^2*x*−*y*−*z*^. Define self-maps *f*, *g*, *h*, *R*, *S*, and *T* on *X* by

(96) $$\begin{array}{c} fx=\ln\left(1+x\right),\;\;gx=\ln\left(1+\frac{x}{2}\right),\\ hx=\ln\left(1+\frac{x}{3}\right),\\ Rx=e^{3x}-1,\;\;Sx=e^{2x}-1,\;\;Tx=e^{6x}-1. \end{array}$$

To prove that (*f*, *g*) is partially weakly *α*-admissible with respect to *R*, let *x* ∈ *X* and *y* ∈ *R* ^−1^ *fx*; that is, *Ry* = *fx*. By the definition of *f* and *R*, we have ln⁡(1 + *x*) = *e* ^3*y*^ − 1. So, *y* = ln⁡(ln⁡(1 + *x*) + 1)/3 and hence

(97) f(x)=ln⁡(1+x)≥ln⁡(1+ln⁡⁡(ln⁡⁡(1+x)+1)3)≥ln⁡(1+ln⁡⁡(ln⁡⁡(1+x)+1)2)=gy.

Therefore, *α*(*fx*, *gy*, *gy*) ≥ 1.

To prove that (*g*, *h*) is partially weakly *α*-admissible with respect to *S*, let *x* ∈ *X* and *y* ∈ *S* ^−1^ *gx*; that is, *Sy* = *gx*. By the definition of *g* and *S*, we have ln⁡(1 + (*x*/2)) = *e* ^2*y*^ − 1. So,

(98) g(x)=ln⁡(1+x2)≥ln⁡(1+ln⁡⁡(ln⁡⁡(1+(x/2))+1)/22)≥ln⁡(1+ln⁡⁡(ln⁡⁡(1+(x/2))+1)/23)=hy.

Therefore, *α*(*gx*, *hy*, *hy*) ≥ 1.

To prove that (*h*, *f*) is partially weakly *α*-admissible with respect to *T*, let *x* ∈ *X* and *y* ∈ *T* ^−1^ *hx*; that is, *Ty* = *hx*. By the definition of *h* and *T*, we have ln⁡(1 + (*x*/3)) = *e* ^6*y*^ − 1. So,

(99) h(x)=ln⁡(1+x3)≥ln⁡(1+ln⁡⁡(ln⁡⁡(1+(x/3))+1)/63)≥ln⁡(1+ln⁡⁡(ln⁡⁡(1+(x/3))+1)/64)=fy.

Therefore, *α*(*hx*, *fy*, *fy*) ≥ 1.

Furthermore, *fX* = *gX* = *hX* = *RX* = *SX* = *TX* = [0, *∞*).

Define *ψ*, *φ* : [0, *∞*)→[0, *∞*) as *ψ*(*t*) = *bt* and *φ*(*t*) = (*b* − 1)*t*, for all *t* ∈ [0, *∞*), where 1 < *b* ≤ 36.

Using the mean value theorem for all *x*, *y*, and *z* with *α*(*Tx*, *Ry*, *Sz*) ≥ 1 we have

(100) ψ(G(fx,gy,hz)) =b|fx−gy|+b|gy−hz|+b|hz−fx| =b|ln⁡(1+x)−ln⁡(1+y2))|  +b|ln⁡(1+y2)−ln⁡(1+z3))|  +b|ln⁡(1+z3)−ln⁡(1+x))| ≤b|x−y2|+b|y2−z3|+b|z3−x| ≤b|6x−3y|6+b|3y−2z|6+b|2z−6x|6 ≤b36|e6x−1−[e3y−1]|+b36|e3y−1−[e2z−1]|  +b36|e2z−1−[e6x−1]| =G(Tx,Ry,Sz) =ψ(G(Tx,Ry,Sz))−φ(G(Tx,Ry,Sz)).

Thus, (6) is true for *M*(*x*, *y*, *z*) = *G*(*Tx*, *Ry*, *Sz*). Therefore, all the conditions of Theorem 19 are satisfied. Moreover, 0 is a coincidence point of all six maps.

## 3. Periodic Point Results

Let *F*(*f*) = {*x* ∈ *X* : *fx* = *x*} be the fixed point set of *f*.

Clearly, a fixed point of *f* is also a fixed point of *f* ^*n*^, for every *n* ∈ *N*; that is, *F*(*f*) ⊂ *F*(*f* ^*n*^). However, the converse is false. For example, the mapping *f* : *R* → *R*, defined by *fx* = (1/2) − *x*, has the unique fixed point 1/4, but every *x* ∈ *R* is a fixed point of *f* ^2^. If *F*(*f*) = *F*(*f* ^*n*^), for every *n* ∈ *N*, then *f* is said to have property *P*. For more details, we refer the reader to [5, 42–45] and the references mentioned therein.

Assume that

(101) M0(x,y,z) ∈{G(x,y,z),   G(x,fy,fy)+G(y,fz,fz)+G(z,fx,fx)6,   G(x,x,fy)+G(y,y,fz)+G(z,z,fx)6,   G(x,fx,fx)+G(y,fy,fy)+G(z,z,fz)6,   G(x,x,fx)+G(y,y,fy)+G(z,fz,fz)6}.

Taking *R* = *I*_*X*_ (the identity mapping on *X*) in Corollary 22, we obtain the following fixed point result.

**Corollary 25 .**

Let (*X*, *G*) be a *G*-complete *G*-metric space. Let *f* : *X* → *X* be a mapping such that *f* is partially weakly *α*-admissible and, for every *x*, *y*, *z* ∈ *X* such that *α*(*x*, *y*, *z*) ≥ 1,

(102) $$\begin{array}{c} \psi \left(G\left(fx,fy,fz\right)\right)\leq \psi \left(M_{0}\left(x,y,z\right)\right)-\varphi \left(M_{0}\left(x,y,z\right)\right), \end{array}$$

where *φ* : [0, *∞*)→[0, *∞*) is an altering distance function. Then, *f* has a fixed point *z* in *X* provided that (a) *f* is continuous or (b) *X* is *α*-regular.

**Theorem 26 .**

Let *X* and *f* be as in Corollary 25. Then *f* has property *P* if *f* is an *α*-dominating map.

**Proof**

From Corollary 25, *F*(*f*) ≠ *∅*. Let *u* ∈ *F*(*f* ^*n*^) for some *n* > 1. We will show that *u* = *fu*. We have *α*(*f* ^*n*−1^ *u*, *f* ^*n*^ *u*, *f* ^*n*^ *u*) ≥ 1, as *f* is *α*-dominating. Using (6), we obtain that

(103) G(u,fu,fu) =G(fnu,fn+1u,fn+1u) =G(ffn−1u,ffnu,ffnu) ≤M(fn−1u,fnu,fnu)−φ(M(fn−1u,fnu,fnu)),

where

(104) M(fn−1u,fnu,fnu)∈{G(fn−1u,fnu,fnu),  (G(fn−1u,ffnu,ffnu)+G(fnu,ffnu,ffnu)   +G(fnu,ffn−1u,ffn−1u))/6,  (G(fn−1u,fn−1u,ffnu)+G(fnu,fnu,ffnu)   +G(fnu,fnu,ffn−1u))/6,  (G(fn−1u,ffn−1u,ffn−1u)+G(fnu,ffnu,ffnu)   +G(fnu,ffnu,ffnu))/6,  (G(fn−1u,fn−1u,ffn−1u)+G(fnu,fnu,ffnu)   +G(fnu,fnu,ffnu))/6}.

If *M*(*f* ^*n*−1^ *u*, *f* ^*n*^ *u*, *f* ^*n*^ *u*) = *G*(*f* ^*n*−1^ *u*, *f* ^*n*^ *u*, *f* ^*n*^ *u*), then, from (103), we have

(105) G(u,fu,fu) ≤G(fn−1u,fnu,fnu)−φ(G(fn−1u,fnu,fnu)).

Starting from *G*(*f* ^*n*−1^ *u*, *f* ^*n*^ *u*, *f* ^*n*^ *u*) and repeating the above process, we get

(106) G(u,fu,fu) ≤G(fn−1u,fnu,fnu)−φ(G(fn−1u,fnu,fnu)) ≤G(fn−2u,fn−1u,fn−1u)−φ(G(fn−2u,fn−1u,fn−u))  −φ(G(fn−1u,fnu,fnu))           ⋮ ≤G(u,fu,fu)  −∑i=0n−1φ(G(fn−(i+1)u,fn−(i)u,fn−(i)u));

which from our assumptions about *φ* implies that

(107) $$\begin{array}{c} G\left(f^{n-(i+1)}u,f^{n-(i)}u,f^{n-(i)}u\right)=0, \end{array}$$

for all 0 ≤ *i* ≤ *n* − 1. Now, taking *i* = *n* − 1, we have *u* = *fu*.

Now, let

(108) M(fn−1u,fnu,fnu) =(G(fn−1u,ffnu,ffnu)+G(fnu,ffnu,ffnu)   +G(fnu,ffn−1u,ffn−1u))/6.

So, we have

(109) G(u,fu,fu)=G(fnu,fn+1u,fn+1u)=G(ffn−1u,ffnu,ffnu)≤(G(fn−1u,fn+1u,fn+1u)+G(fnu,fn+1u,fn+1u)  +G(fnu,fnu,fnu))/6 −φ((G(fn−1u,fn+1u,fn+1u)+G(fnu,fn+1u,fn+1u)    +G(fnu,fnu,fnu))/6)≤(G(fn−1u,fnu,fnu)+2G(fnu,fn+1u,fn+1u)+0)/6 −φ((G(fn−1u,fn+1u,fn+1u)    +G(fnu,fn+1u,fn+1u)+0)/6);

that is,

(110) G(u,fu,fu) =G(fnu,fn+1u,fn+1u) ≤G(fn−1u,fnu,fnu)  −3φ((G(fn−1u,fn+1u,fn+1u)+G(fnu,fn+1u,fn+1u)+0)/6).

Repeating the above process, we get

(111) G(fn−1u,fnu,fnu) ≤4G(fn−1u,fnu,fnu) ≤G(fn−2u,fn−1u,fn−1u)  −6φ(G(fn−2u,fnu,fnu)+G(fn−1u,fnu,fnu)6).

From the above inequalities, we have

(112) G(u,fu,fu) ≤G(u,fu,fu)  −6∑i=0n−1φ((G(fn−(i+1)u,fn−(i−1)u,fn−(i−1)u)+G(fn−(i)u,fn−(i−1)u,fn−(i−1)u))/6).

Therefore,

(113) ∑i=0n−1φ((G(fn−(i+1)u,fn−(i−1)u,fn−(i−1)u)   +G(fn−(i)u,fn−(i−1)u,fn−(i−1)u))/6)=0,

which from our assumptions about *φ* implies that

(114) G(fn−(i+1)u,fn−(i−1)u,fn−(i−1)u) =G(fn−(i)u,fn−(i−1)u,fn−(i−1)u)=0

for all 0 ≤ *i* ≤ *n* − 1. Now, taking *i* = *n* − 1, we have *u* = *fu*.

In other three cases, the proof will be done in a similar way.

## 4. Results in Ordered *G*-Metric Spaces

Fixed point theorems for monotone operators in ordered metric spaces are widely investigated and have found various applications in differential and integral equations (see [46–48], and references therein). As an application of our results, we derive some new coincidence point and common fixed point theorems for partially weakly increasing contractions which generalize many results in the literature.

**Definition 27 (see [49]).**

Let (*X*, ⪯, *G*) be a partially ordered *G*-metric space. We say that *X* is regular if and only if the following hypothesis holds.

For any nondecreasing sequence {*x*_*n*_} in *X* such that *x*_*n*_ → *z* as *n* → *∞*, it follows that *x*_*n*_⪯*z* for all *n* ∈ *N*.

**Definition 28 (see [49]).**

Let (*X*, ⪯) be a partially ordered set and *f*, *g*, *h* : *X* → *X* given mappings such that *fX*⊆*hX* and *gX*⊆*hX*. We say that *f* and *g* are weakly increasing with respect to *h* if and only if for all *x* ∈ *X*, *fx*⪯*gy*, for all *y* ∈ *h* ^−1^(*fx*), and *gx*⪯*fy*, for all *y* ∈ *h* ^−1^(*gx*).

If *f* = *g*, we say that *f* is weakly increasing with respect to *h*.

**Definition 29 (see [49]).**

Let (*X*, ⪯) be a partially ordered set and *f* and *g* two self-maps on *X*. An ordered pair (*f*, *g*) is said to be partially weakly increasing with respect to *h* if *fx*⪯*gy*, for all *y* ∈ *h* ^−1^(*fx*).

If *h* = *I* (the identity mapping on *X*), then the previous definition reduces to the weakly increasing mapping [50] (also see [51, 52]).

Note that a pair (*f*, *g*) is weakly increasing with respect to *h* if and only if ordered pairs (*f*, *g*) and (*g*, *f*) are partially weakly increasing with respect to it.

Let (*X*, ⪯, *G*) be a partially ordered set and let

(115) $$\begin{array}{c} \begin{array}{c} \alpha \left(x,y,z\right)=\{\begin{array}{cc} 1, & x⪯y⪯z,\\ 0, & \text{otherwise}. \end{array} \end{array} \end{array}$$

**Theorem 30 .**

Let (*X*, ⪯, *G*) be a partially ordered *G*-complete *G*-metric space. Let *f*, *g*, *h*, *R*, *S*, *T* : *X* → *X* be six mappings such that *f*(*X*)⊆*R*(*X*), *g*(*X*)⊆*S*(*X*), and *h*(*X*)⊆*T*(*X*). Suppose that, for every three elements *Tx*⪯*Ry*⪯*Sz*, one has

(116) $$\begin{array}{c} \psi \left(G\left(fx,gy,hz\right)\right)\leq \psi \left(M\left(x,y,z\right)\right)-\varphi \left(M\left(x,y,z\right)\right), \end{array}$$

where *ψ*, *φ* : [0, *∞*)→[0, *∞*) are altering distance functions. Let *f*, *g*, *h*, *R*, *S*, and *T* be continuous, the pairs (*f*, *T*), (*g*, *R*), and (*h*, *S*) compatible, and the pairs (*f*, *g*), (*g*, *h*), and (*h*, *f*) partially weakly increasing with respect to *R*, *S*, and *T*, respectively. Then, the pairs (*f*, *T*), (*g*, *R*), and (*h*, *S*) have a coincidence point *z* in *X*. Moreover, if *Tz*⪯*Rz*⪯*Sz*, then *z* is a coincidence point of *f*, *g*, *h*, *R*, *S*, and *T*.

**Theorem 31 .**

Let (*X*, ⪯, *G*) be a regular partially ordered *G*-metric space, *f*, *g*, *h*, *R*, *S*, *T* : *X* → *X* six mappings such that *f*(*X*)⊆*R*(*X*), *g*(*X*)⊆*S*(*X*), and *h*(*X*)⊆*T*(*X*), and *RX*, *SX*, and *TX* *G*-complete subsets of *X*. Suppose that, for elements *Tx*⪯*Ry*⪯*Sz*, one has

(117) $$\begin{array}{c} \psi \left(G\left(fx,gy,hz\right)\right)\leq \psi \left(M\left(x,y,z\right)\right)-\varphi \left(M\left(x,y,z\right)\right), \end{array}$$

where *ψ*, *φ* : [0, *∞*)→[0, *∞*) are altering distance functions. Then, the pairs (*f*, *T*), (*g*, *R*), and (*h*, *S*) have a coincidence point *z* in *X* provided that the pairs (*f*, *T*), (*g*, *R*), and (*h*, *S*) are weakly compatible and the pairs (*f*, *g*), (*g*, *h*), and (*h*, *f*) are partially weakly increasing with respect to *R*, *S*, and *T*, respectively. Moreover, if *Tz*⪯*Rz*⪯*Sz*, then *z* ∈ *X* is a coincidence point of *f*, *g*, *h*, *R*, *S*, and *T*.
